# Influence of Material Properties on Rate of Resorption of Two Bone Graft Materials after Sinus Lift Using Radiographic Assessment

**DOI:** 10.1155/2012/737262

**Published:** 2012-07-31

**Authors:** Fawzi Riachi, Nada Naaman, Carine Tabarani, Nayer Aboelsaad, Moustafa N. Aboushelib, Antoine Berberi, Ziad Salameh

**Affiliations:** ^1^Department of Oral Surgery, Faculty of Dental Medicine, Saint Joseph University, P.O. Box 17-5208, Beirut 1104-2020, Lebanon; ^2^Department of Periodontology, Faculty of Dental Medicine, Saint Joseph University, P.O. Box 17-5208, Beirut 1104-2020, Lebanon; ^3^Periodontology Department, Faculty of Dentistry, Beirut Arab University, P.O. Box 115020 Rial el Solh, 1107 Beirut, Lebanon; ^4^Dental Biomaterials Department, Faculty of Dentistry, Alexandria University, Alexandria, Egypt; ^5^Research Department, School of Dental Medicine, Lebanese University, P.O. Box 4 Hadath, Lebanon

## Abstract

*Purpose*. The aim of this study was to investigate the influence of chemical and physical properties of two graft materials on the rate of resorption. *Materials and Methods*. Direct sinus graft procedure was performed on 22 patients intended for implant placement. Two types of graft materials were used (Bio-Oss and Cerabone) and after 8 months healing time the implants were inserted. Radiographic assessment was performed over the period of four years. Particle size, rate of calcium release, and size and type of crystal structure of each graft were evaluated. *Results.* The average particle size of Bio-Oss (1 mm) was much smaller compared to Cerabone (2.7 mm). The amount of calcium release due to dissolution of material in water was much higher for Bio-oss compared to Cerabone. X-ray image analysis revealed that Bio-Oss demonstrated significantly higher volumetric loss (33.4 ± 3.1%) of initial graft size compared to Cerabone (23.4 ± 3.6%). The greatest amount of vertical loss of graft material volume was observed after one year of surgery. *Conclusion*. The chemical and physical properties of bone graft material significantly influence resorption rate of bone graft materials used for sinus augmentation.

## 1. Introduction

Maxillary sinus augmentation and placement of dental implants is a well-established technique for functional and esthetic rehabilitation of partially or completely edentulous patients with severe maxillary atrophy. Sinus pneumatization, together with poor bone quality, is one of the most challenging circumstances in implantology, a condition that will restrict implant placement in such areas. When this situation occurs, bone grafts can be used to correct bone deficits, allowing the placement of implants of adequate length and width [1]. The first report about maxillary sinus floor augmentation for placement of implants was published by Boyne and James [2], while Tatum [3] first described two techniques with a sinus approach from the alveolar crest and lateral wall in maxillary and sinus implant reconstruction.

There are diverse choices of graft materials available for replacing lost bone through atrophy, trauma, congenital or pathological processes. These graft materials include: intra or extraoral autologous bone, heterologous grafts, alloplastic grafts, xenografts or a combination of these [4]. In general, the success of a bone graft is measured in terms of its capacity to withstand the conditions of tension and mechanical deformation to which it is subjected. The interactions between graft material and healing processes at the host site have a direct influence on the pattern, rate, and quality of new bone formation. Successful grafts are those that undergo revascularization and substitution of the graft material by host bone, without suffering a significant loss of mechanical strength or graft volume [5, 6].

Clinical and histomorphometric studies done on autografts, bovine hydroxyapatite (Bio-Oss, Geistlich), a xenograft and *β*-tricalciumphosphate (Cerasorb, Curasan), an alloplast, prove that all these grafting materials are biocompatible, osseoconductive and can be used successfully in conjunction during implant rehabilitation [7, 8]. However, rate of resorption of these materials is dependent on their chemical and physical properties. Frenken et al. [9] evaluated the quantity and quality of bone formed in sinus augmentations using a synthetic material: biphasic calcium phosphate consisting of a combination of 60% hydroxyapatite and 40% *β*-tricalcium phosphate. Histological findings reported differences in the amount of newly formed bone used with each material.

 The aim of this study was to evaluate the influence of chemical and physical properties of two types of bone graft materials on the rate of resorption after placement in sinus lift procedure over a period of four years.

## 2. Materials and Methods

 This study was conducted in coherence with the Helsinki agreement for research on humans and the study design was approved by the Institutional Review Board and Independent Ethics Committee of the Faculty of Dental Medicine, Saint Joseph University, Beirut, Lebanon. Signed informed consent forms were obtained for all participants in the study.

 Two xenograft materials prepared by deproteinizing technique (Bio-Oss, Geistlich Sons Ltd., Wolhusen, Switzerland) or high temperature decalcified freeze-dried (Cerabone, Botiss Dental, Berlin, Germany) were selected for this study.

### 2.1. Characterization of the Graft Materials

The particle size of each graft material was calculated using particle size analyzer (Partica LA-950V2, Horiba Scientific, Kyoto, Japan), and average particle size and distribution were calculated from 5 different batches for each material.

Crystal structure and size of crystals were calculated using X-ray diffraction (XRD) technique. 5 gram of each material was finely ground, dried, and homogenously dispersed on the measuring table of the machine (Bruker AXS, D8 Advance, Bruker AXS GmbH, Karlsruhe, Germany, 10°/min, 2 *θ* from 5° to 60°). The phase composition was checked using Joint Committee on Powder Diffraction standards. Crystallite size analysis was calculated using the peak broadening of XRD reflection that is used to estimate the crystallite size (in a direction perpendicular to the crystallographic plane) using the following formula:

(1) Xs=0.9λ(FWHM×cos⁡θ),

where *Xs* is the crystallite size in nanometer, *λ* is the wavelength of X-ray beam in nanometer (*λ* = 0.15406 nm for standard detectors), and FWHM is the full width at half maximum for the diffraction angle (2*θ* = 25.9° peak was selected related to (002) Miller's plane family).

 Solubility of graft material in demineralized water was evaluated using atomic absorption spectrophotometer (WFX-210, RayLeigh, BRAIC, China). Calcium and phosphorous detectors were calibrated in standard solution before each reading. 0.25 gram of each material was immersed in 100 mL of double purified water and the amount of calcium dissolution was measured every week for a period of six months.

Patients received detailed explanations of the difficulties and complications that could take place during the surgery and all patients agreed before the surgery. All of the 22 consenting patients were examined and medically compromised and uncooperative cases were excluded from the study.

### 2.2. Sinus Lift Technique

**Figure 1:**
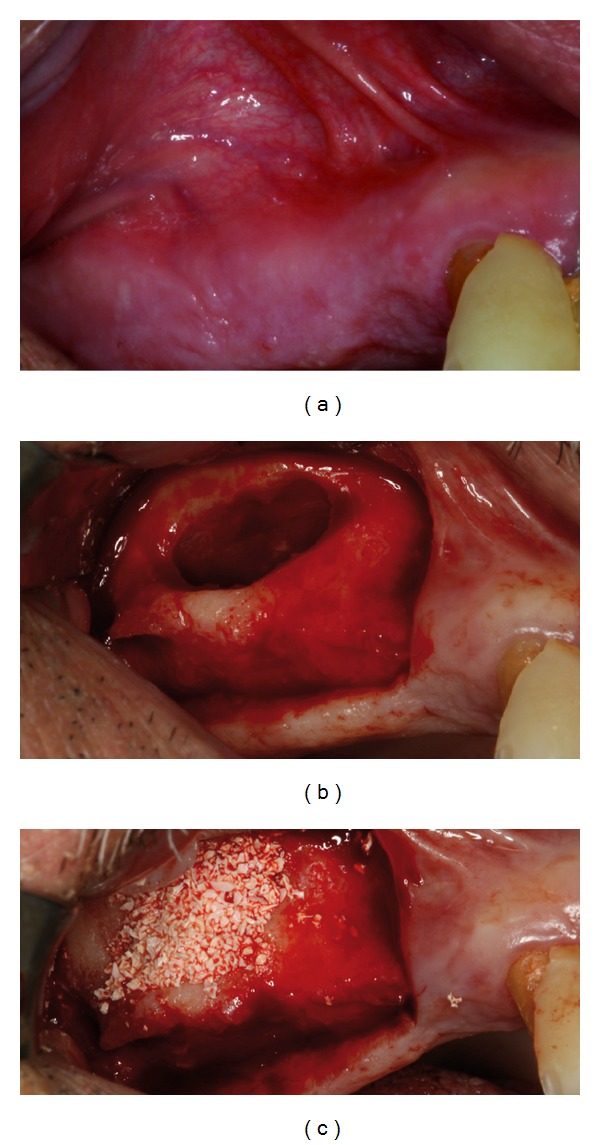
Site before exposure (a), direct exposure of lateral sinus wall (b), and filling of sinus with the selected grafting material (c).

Local anesthesia was administered (2% lidocaine containing 1 : 100,000 epinephrine) and a horizontal incision was made along on the crestal bone in the edentulous area and then vertical incisions were made to elevate the mucoperiosteal flap. After elevation of a full-thickness mucoperiosteal flap, access was gained to the anterior bony wall of the sinus. The lateral bony wall of the sinus was cut by using a small diamond bur. All the cortical bone was removed up to the sinus membrane. After elevation of the membrane, the sinus cavity was then packed with either of the selected materials Figures 1(a), 1(b), and 1(c). An absorbable collagen membrane (Bio-Gide, Geistlich Pharma AG, Wolhusen, Switzerland) was then placed on the graft to avoid migration of the graft and invasion of soft tissues. After the surgery, patients were prescribed 625 mg of antibiotic (Augmentin, GlaxoSmithKline, United Kingdom) twice a day for a week and advised to rinse their mouths daily with Chlorhexidine Gluconate Oral Rinse 0.12% (PerioGard, Colgate-Palmolive, United Kingdom) during healing period. The patients were examined 1 week after surgery when the sutures were removed. All patients were checked regularly to verify healing. After a healing period of 8 months, all implants (NobelReplace, Nobel Biocare, Kloten, Switzerland) were placed by one expert oral surgeon. The choice of the implant length was based on the postpanoramic X-rays after the sinus lift surgery.

### 2.3. Measurement of Graft Height

Height of graft material was measured at the following intervals:

- 1st measurement: right after the implantation (baseline),
- 2nd measurement: after 8 month at time of implant placement,
- 3rd measurement: one year after implant placement,
- 4th measurement: four years after implant placement.

The implant length, alveolar crest, the original base line of the sinus floor, and the final graft height were traced by superimposition of the panoramic images. Two fixed measurement points were evaluated using image analysis software (Cell A, Olympus, Germany) to the accuracy of 1 um. [10]. Implant length was used to correct for any magnification errors.

### 2.4. Statistical Analysis

Data were analyzed using computer statistical program software (SPSS 18.0, SPSS Inc, Chicago, Il, USA) to evaluate the resorption rate of graft material with time and the differences between graft materials (means and standard deviations). Changes in graft volume at different time intervals were analyzed using Student's *t*-test (*α* = 0.05).

## 3. Results

**Figure 2:**
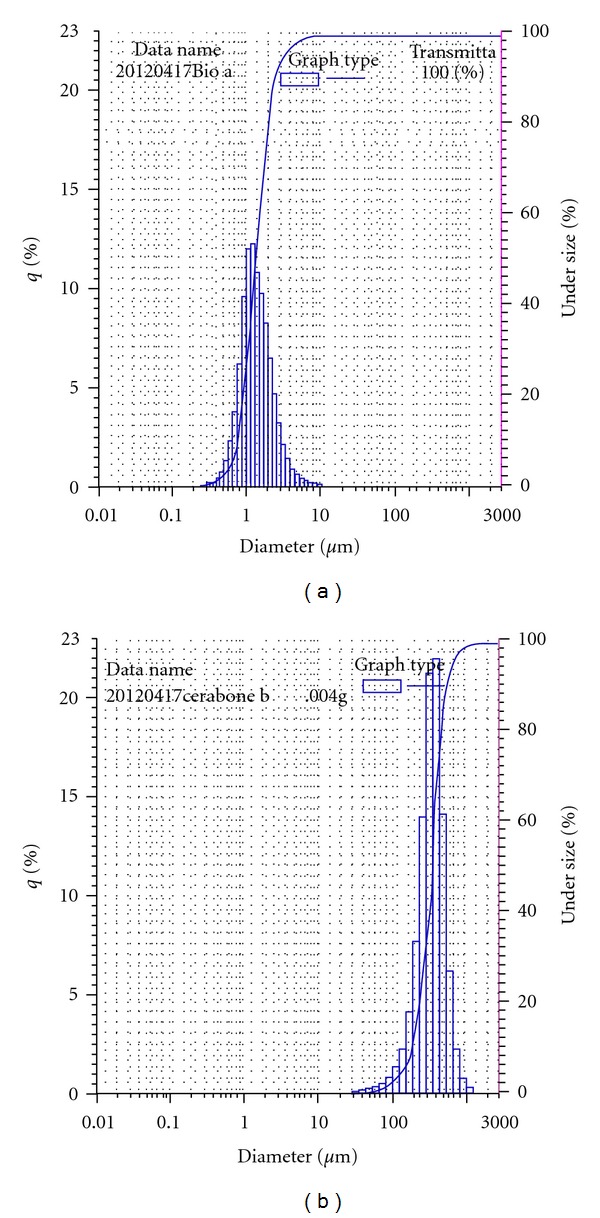
(a) Average particle size and distribution of Bio-Oss, (b) average particle size and distribution of Cerabone.

**Figure 3:**
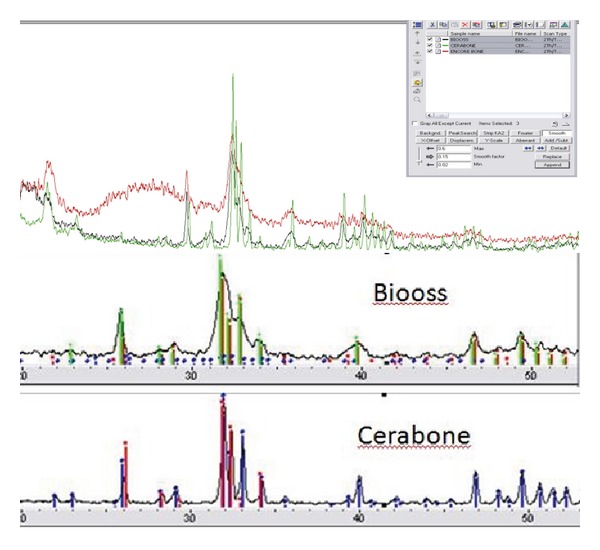
XRD analysis of Bio-Oss (red) and Cerabone (green) in relation to natural hydroxylapatite (black).

The average particle size of Bio-Oss (1 mm) was much smaller compared to Cerabone (2.7 mm), Figures 2(a) and 2(b). X-ray diffraction analysis revealed typical structure of hydroxylapatite for both materials. The crystallite size was smaller for Bio-Oss (41.7 nm at 25.86 diffraction angle) compared to Cerabone (53.2 nm at 25.95 diffraction angle), Figure 3.

**Figure 4:**
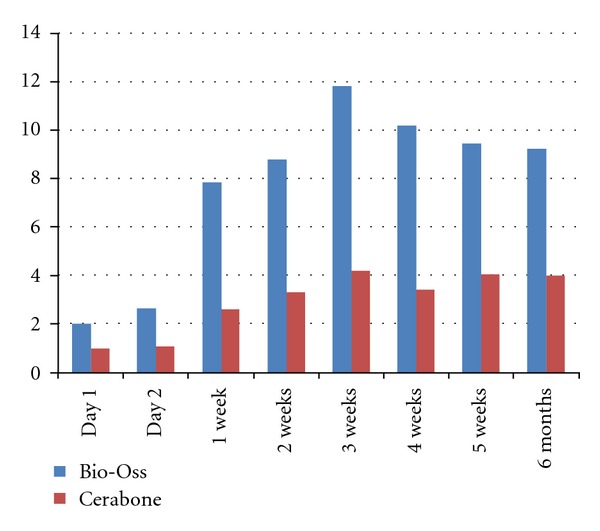
Calcium release (mg/g) at different time intervals. Release rate was almost constant after 2 months.

The amount of calcium release due to dissolution of the material in water was much higher for Bio-Oss compared to Cerabone. This observation was marked in the first 6 weeks after which dissolution rate of calcium ions reaches a fixed rate for both materials, Figure 4.

**Figure 5:**
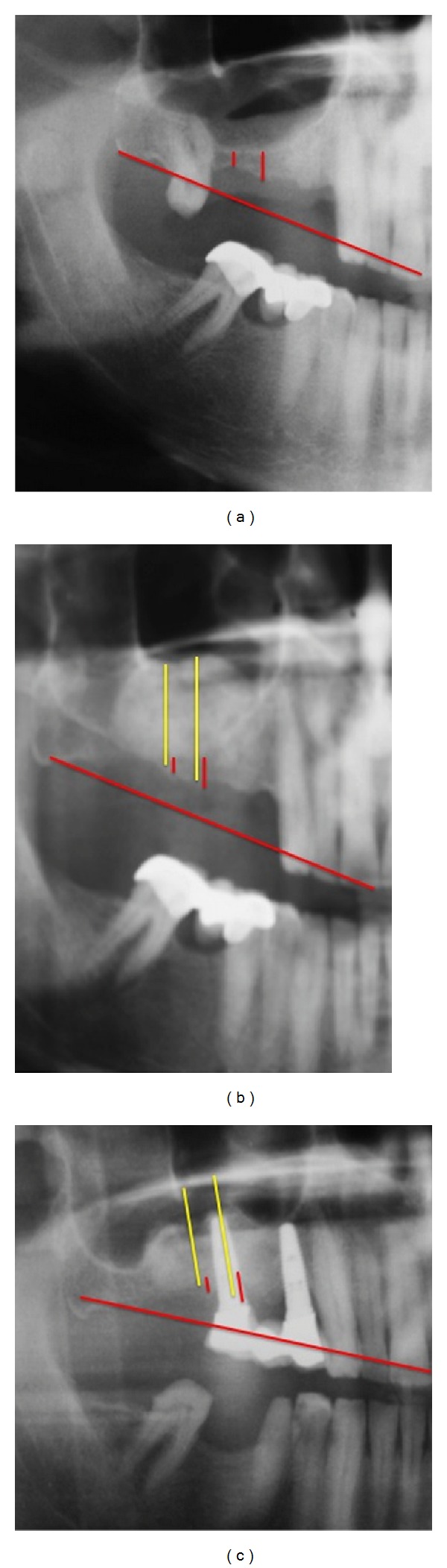
Panoramic X-ray with fixed measuring points at base line (a), after grafting procedure using Bio-Oss after 8-month healing time (b), and after four years of implant placement (c).

**Figure 6:**
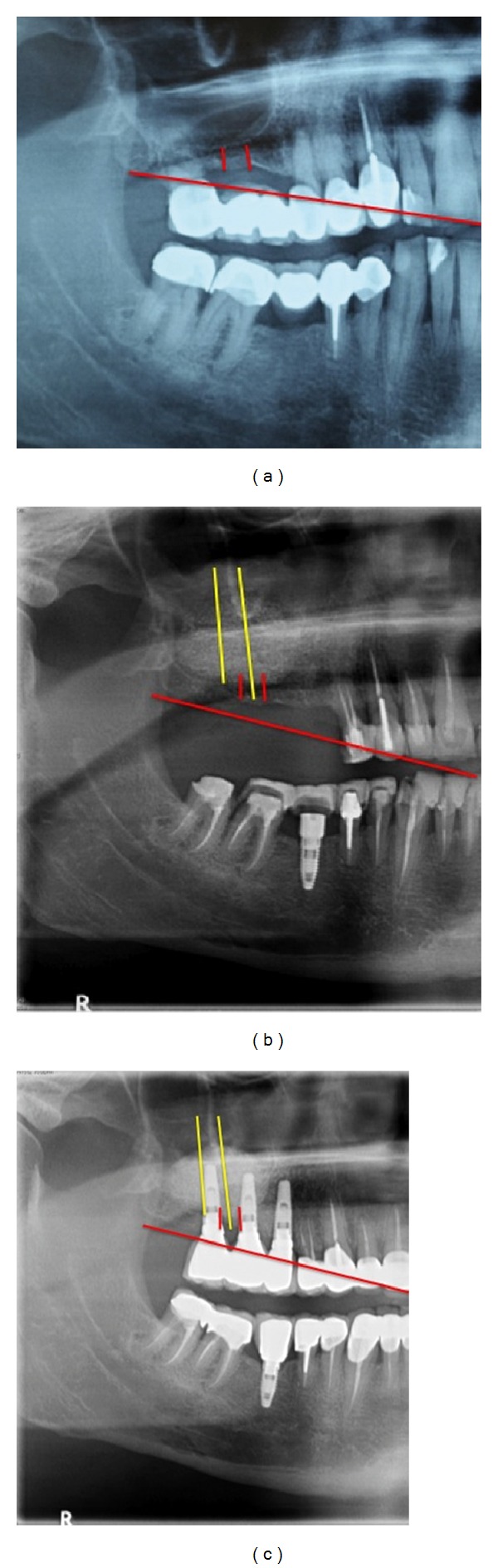
Panoramic X-ray with fixed measuring points at base line (a), after grafting procedure using Cerabone (b), and four years of implant placement (c).

Four implants failed after 6 months from insertion time due to lack of adequate initial stability, these cases were replaced with new cases. All patients demonstrated adequate healing after grafting surgery without complications. X-ray image analysis revealed that Bio-Oss demonstrated significantly higher (*t* = 7.25, *P* < 0.001) volumetric loss (33.4 ± 3.1%, volumetric loss of total graft height after 4 years) compared to Cerabone (23.4 ± 3.6%). The greatest amount of vertical loss of graft volume was observed after one year of graft surgery (55–65% of total bone loss), which decreased almost to 10–12% per year later on for both materials (*P* < 0.06), Figures 5 and 6. After four years from implant placement, it was observed that the height of Bio-Oss bone graft was located at level of implant apex while this finding was not reported for Cerabone.

## 4. Discussion

 Numerous allogenic or alloplastic materials have been used alone or in combination with autogenous bone for sinus augmentation. Many researchers showed that these materials could be as effective as autologous bone [11–19]. Histologic evidence generated by studies of mature grafts and the excellent survival rates of implants inserted in them have led to the realization that these nonautogenous graft materials may be considered an excellent option [9, 13, 15, 20–23].

Moy et al. [24] reported 59.4 ± 18.0% new bone formation and 40.5 ± 17.9% connective tissue in the histomorphometric analysis of sinus augmented with chin bone after six-month healing time, The quality of newly formed bone was superior when compared to bovine hydroxyapatite and *β*-tricalciumphosphate, as it was composed of about 80% lamellar mature in nature. Another histomorphometric study [25] using Bio-Oss showed 28% mature bone, 44% connective tissue, and 28% bovine hydroxyapatite (BHA) particles in a period of 6 months from 20 sinus lifts done in 15 patients.

A ten-year follow-up study [26] from 36 sinus grafts reported 29.8 ± 2.5% new bone formation in the first 8 months, 69.7 ± 2.6% in the next one year, and by the end of the study it was 86.7 ± 2.84%. The study proved that the rate of resorption of the graft material, BHA, was 3.55% per month in the initial 2 years and then the value reached a mean value of 0.58% per month in the next 8 years that is close to the findings of the present study. A total volume loss after 4 years was 34% for Bio-Oss and 22% for Cerabone accounting for an average monthly volume loss of 0.69% for Bio-Oss and 0.5% per month for Cerabone.

Although BHA is considered to be a resorbable material, it is not clear from the literature if the graft particles will undergo resorption and will eventually be replaced with autogenous bone. Moreover the bone found in conjunction with the BHA particles was mainly woven [27]. Based on the data observed in the present study, Bio-Oss has smaller particle size (1 mm average particle size compared to 2.7 mm for Cerabone) resulting in significantly higher surface area, higher calcium release rate (9.8 mg/g), and smaller crystallite size (41.7 nm at 25.86 diffraction angle) compared to 53.2 nm at 25.95 diffraction angle for Cerabone. These minor differences were associated with significantly higher resorption rate of the initial graft volume observed for Bio-Oss material.

Studies [28, 29] using *β*-tricalciumphosphate (*β*-TCP) in sinus augmentation show around 29% new bone formation after 6 months healing time. When an osseoinductive factor like platelet rich plasma (PRP) was mixed with *β*-TCP, the osseous regenerating capacity was increased to 38%. Nevertheless, a resorption rate of 32–43% was reported; type and quality of crystal content of graft material is a dominant factor-controlling rate of resorption.

 A very recent study [30] performed an ultrastructural study on bone-to-biomaterial interface and biomaterial mineral degradation in retrieved bone biopsies following maxillary sinus augmentation using bovine xenografts (Endobon). Scanning electron microscopy revealed that newly formed bone was closely attached to the xenograft. Elemental analysis showed a significantly high Ca/P ratio in the residual biomaterials (3.031 ± 0.104) compared with the interface (2.908 ± 0.115) and new bone (2.889 ± 0.113), which suggests that there may be a gradual diffusion of Ca ions from the biomaterial into the newly forming bone at the interface as part of the biomaterial's resorption process. These findings are in direct agreement with the calcium release rate observed in the present study. Under the influence of body fluids and with consideration to flaw dynamics of blood, a higher calcium release rate is expected inside the sinus due to washing-off effect of the released ions, Figure 4.

 Jensen et al. [31] reported that the types of graft materials influence the resorption rate of bone which was 1.8 mm in an autograft, 2.1 mm in a demineralized allograft, 0.9 mm in an alloplast, and 0.8 mm in an autograft mixed with alloplast. Histologic reviews of sinus lift procedures [32] with different types of graft material reported height reduction with all graft materials. Furthermore, in 90% of cases, the graft materials were positioned superior to the apex of the implant, which is in agreement with the findings of this study. The cases grafted with Bio-Oss ended with graft resorption ending at apex of integrated implants after four-year service time, meanwhile at least 3 mm of new bone remained on top of implants inserted in Cerabone graft. Hatano et al. [10] reported that graft materials were reduced with a statistically significant amount during 2 to 3 years after a sinus lift, while other study [33] observed that the force loading on dental implants caused graft height to be sustained at a consistent level.

These results should be interpreted cautiously considering the study's reduced sample size. Further in vitro and in vivo studies should be conducted to validate the results of the present study.

## 5. Conclusions

Within limitations of this study, the physical and chemical properties of bone graft material have significant influence on rate of resorption after sinus lift procedure intended for implant placement. Careful consideration of graft properties might enhance clinical performance.

## References

[1] Aguirre Zorzano LA, Rodríguez Tojo MJ, Aguirre Urizar JM (2007). Maxillary sinus lift with intraoral autologous bone and B—tricalcium phosphate: histological and histomorphometric clinical study. *Medicina Oral, Patologia Oral y Cirugia Bucal*.

[2] Boyne PJ, James RA (1980). Grafting of the maxillary sinus floor with autogenous marrow and bone. *Journal of Oral Surgery*.

[3] Tatum H (1986). Maxillary and sinus implant reconstructions. *Dental Clinics of North America*.

[4] Misch CE, Dietsh F (1993). Bone-grafting materials in implant dentistry. *Implant Dentistry*.

[5] Guarnieri R, Grassi R, Ripari M, Pecora G (2006). Maxillary sinus augmentation using granular calcium sulfate (Surgiplaster Sinus): radiographic and histologic study at 2 years. *International Journal of Periodontics and Restorative Dentistry*.

[6] Park JB (2010). Radiographic follow-up evaluation of sinus augmentation with deproteinized bovine bone and implant installation after loading. *Indian Journal of Dental Research*.

[7] Norton MR, Odell EW, Thompson ID, Cook RJ (2003). Efficacy of bovine bone mineral for alveolar augmentation: a human histologic study. *Clinical Oral Implants Research*.

[8] Zerbo IR, Zijderveld SA, De Boer A (2004). Histomorphometry of human sinus floor augmentation using a porous *β*-tricalcium phosphate: a prospective study. *Clinical Oral Implants Research*.

[9] Frenken JWFH, Bouwman WF, Bravenboer N, Zijderveld SA, Schulten EAJM, Ten Bruggenkate CM (2010). The use of Straumann Bone Ceramic in a maxillary sinus floor elevation procedure: a clinical, radiological, histological and histomorphometric evaluation with a 6-month healing period. *Clinical Oral Implants Research*.

[10] Hatano N, Shimizu Y, Ooya K (2004). A clinical long-term radiographic evaluation of graft height changes after maxillary sinus floor augmentation with a 2:1 autogenous bone/xenograft mixture and simultaneous placement of dental implants. *Clinical Oral Implants Research*.

[11] Blus C, Szmukler-Moncler S, Salama M, Salama H, Garber D (2008). Sinus bone grafting procedures using ultrasonic bone surgery: 5-Year experience. *International Journal of Periodontics and Restorative Dentistry*.

[12] Wallace SS, Froum SJ, Cho SC (2005). Sinus augmentation utilizing anorganic bovine bone (Bio-Oss) with absorbable and nonabsorbable membranes placed over the lateral window: histomorphometric and clinical analyses. *International Journal of Periodontics and Restorative Dentistry*.

[13] Lee YM, Shin SY, Kim JY, Kye SB, Ku Y, Rhyu IC (2006). Bone reaction to bovine hydroxyapatite for maxillary sinus floor augmentation: histologic results in humans. *International Journal of Periodontics and Restorative Dentistry*.

[14] Simion M, Fontana F, Rasperini G, Maiorana C (2004). Long-term evaluation of osseointegrated implants placed in sites augmented with sinus floor elevation associated with vertical ridge augmentation: a retrospective study of 38 consecutive implants with 1- to 7-year follow-up. *International Journal of Periodontics and Restorative Dentistry*.

[15] Lekholm U, Wannfors K, Isaksson S, Adielsson B (1999). Oral implants in combination with bone grafts: a 3-year retrospective multicenter study using the Brånemark implant system. *International Journal of Oral and Maxillofacial Surgery*.

[16] Lee JH, Jung UW, Kim CS, Choi SH, Cho KS (2008). Histologic and clinical evaluation for maxillary sinus augmentation using macroporous biphasic calcium phosphate in human. *Clinical Oral Implants Research*.

[17] Maiorana C, Sigurtà D, Mirandola A, Garlini G, Santoro F (2006). Sinus elevation with alloplasts or xenogenic materials and implants: an up-to-4-year clinical and radiologic follow-up. *International Journal of Oral and Maxillofacial Implants*.

[18] Chiapasco M, Zaniboni M, Rimondini L (2008). Dental implants placed in grafted maxillary sinuses: a retrospective analysis of clinical outcome according to the initial clinical situation and a proposal of defect classification. *Clinical Oral Implants Research*.

[19] Martos Díaz P, Naval Gías L, Sastre Pérez J (2007). Sinus elevation by in situ utilization of bone scrapers: technique and results. *Medicina Oral, Patologia Oral y Cirugia Bucal*.

[20] Yamamichi N, Itose T, Neiva R, Wang HL (2008). Long-term evaluation of implant survival in augmented sinuses: a case series. *International Journal of Periodontics and Restorative Dentistry*.

[21] González García R, Naval Gías L, Muñoz Guerra MF, Sastre Pérez J, Rodríguez Campo FJ, Gil-Díez Usandizaga JL (2005). Preprosthetic and implantological surgery in patients with severe maxillary atrophy. *Medicina Oral, Patologia Oral y Cirugia Bucal*.

[22] Hallman M, Sennerby L, Lundgren S (2002). A clinical and histologic evaluation of implant integration in the posterior maxilla after sinus floor augmentation with autogenous bone, bovine hydroxyapatite, or a 20:80 mixture. *International Journal of Oral and Maxillofacial Implants*.

[23] Kahnberg KE, Vannas-Löfqvist L (2008). Sinus lift procedure using a 2-stage surgical technique: i. Clinical and radiographic report up to 5 years. *International Journal of Oral and Maxillofacial Implants*.

[24] Moy PK, Lundgren S, Holmes RE (1993). Maxillary sinus augmentation: histomorphometric analysis of graft materials for maxillary sinus floor augmentation. *Journal of Oral and Maxillofacial Surgery*.

[25] Valentini P, Abensur D (1997). Maxillary sinus floor elevation for implant placement with demineralised freezed-dried bone and bovine bone (Bio-Oss). *International Journal of Periodontics and Restorative Dentistry*.

[26] Sartori S, Silvestri M, Forni F, Cornaglia AI, Tesei P, Cattaneo V (2003). Ten-year follow-up in a maxillary sinus augmentation using anorganic bovine bone (Bio-Oss). A case report with histomorphometric evaluation. *Clinical Oral Implants Research*.

[27] Schou S, Holmstrup P, Jørgensen T (2003). Anorganic porous bovine-derived bone mineral (Bio-Oss®) and ePTFE membrane in the treatment of peri-implantitis in cynomolgus monkeys. *Clinical Oral Implants Research*.

[28] Reinhardt C, Kreusser B (2000). Retrospective study dental implantation with sinus lift and Cerasorb augmentation. *Dental Implantology*.

[29] Cabezas-Mojón J, Barona-Dorado C, Gómez-Moreno G, Fernández-Cáliz F, Martínez-González J-M (2012). Meta-analytic study of implant survival following sinus augmentation. *Medicina Oral, Patologia Oral y Cirugia Bucal*.

[30] Ramírez-Fernández MP, Calvo-Guirado JL, Delgado-Ruiz RA, Maté-Sánchez del Val JE, Negri B, Peñarrocha Diago M Ultrastructural study by backscattered electron imaging and elemental microanalysis of biomaterial-to-bone interface and mineral degradation of bovine xenografts in maxillary sinus floor elevation.

[31] Jensen OT, Shulman LB, Block MS, Lacono VJ (1998). Report of the sinus consensus conference of 1996. *International Journal of Oral and Maxillofacial Implants*.

[32] Nyström E, Kahnberg KE, Albrektsson T (1993). Treatment of the severely resorbed maxillae with bone graft and titanium implants: histologic review of autopsy specimens. *International Journal of Oral & Maxillofacial Implants*.

[33] Listrom RD, Symington JM (1988). Osseointegrated dental implants in conjunction with bone grafts. *International Journal of Oral & Maxillofacial Surgery*.

